# Comment on: Predictive value of baseline serum carbohydrate antigen 19-9 level on treatment effect of neoadjuvant chemoradiotherapy in patients with resectable and borderline resectable pancreatic cancer in two randomized trials

**DOI:** 10.1093/bjs/znae085

**Published:** 2024-04-03

**Authors:** Patrick W Underwood, Jordan M Cloyd

**Affiliations:** Department of Surgery, The Ohio State University Wexner Medical Center, Columbus, Ohio, USA; Department of Surgery, The Ohio State University Wexner Medical Center, Columbus, Ohio, USA

*Dear Editor*

The recent study by Doppenberg *et al*.^1^, regarding the use of baseline carbohydrate antigen 19-9 (CA19-9) as a predictive biomarker for patients undergoing neoadjuvant therapy for pancreatic ductal adenocarcinoma (PDAC), is of great interest. Neoadjuvant therapy is increasingly utilized for patients with localized PDAC, but evidence from existing RCTs has been mixed. This has highlighted the urgent need for predictive biomarkers to help guide personalized treatment. In a post-hoc analysis of two RCTs of patients with resectable or borderline resectable PDAC undergoing neoadjuvant chemoradiation *versus* upfront surgery, Doppenberg *et al*.^1^ found that neoadjuvant chemoradiation led to improved overall survival (OS) among those with a baseline CA19-9 less than or equal to 500 units/ml (HR 0.63, 95% c.i. 0.42 to 0.93), but not among those with CA19-9 greater than 500 units/ml (HR 0.82, 95% c.i. 0.45 to 1.49).

Doppenberg *et al*.^1^ are to be congratulated on these exciting findings. However, it is felt that their importance may actually have been undersold. Despite showing that baseline CA19-9 dichotomized patients with PDAC into two distinct cohorts who did and did not respond to neoadjuvant chemoradiation, Doppenberg *et al*.^1^ concluded that ‘it was not a predictive factor for the treatment effect of neoadjuvant treatment on OS’. We disagree! To the best of our knowledge, this represents the first evidence of a ‘predictive’ biomarker for guiding neoadjuvant therapy in PDAC. While neoadjuvant chemoradiation did not lead to improved outcomes compared with upfront surgery in patients with CA19-9 greater than 500 units/ml, these patients would probably be better served receiving neoadjuvant systemic chemotherapy. In light of the recent PREOPANC-2 trial, suggesting similar outcomes between neoadjuvant FOLFIRINOX (a combination of fluorouracil with leucovorin, oxaliplatin, and irinotecan) and gemcitabine-based chemoradiation, it would be highly informative if similar post-hoc analyses could be conducted for these cohorts. Once again, Doppenberg *et al*.^1^ are to be congratulated on this outstanding work and the incorporation of CA19-9 as a biomarker in future prospective clinical trials is urged.
